# Isolation and Whole-Genome Sequencing of 12 Mushroom-Associated Bacterial Strains: an Inquiry-Based Laboratory Exercise in a Genomics Course at the Rochester Institute of Technology

**DOI:** 10.1128/MRA.01457-19

**Published:** 2020-02-06

**Authors:** Narayan H. Wong, Andrew J. Rosato, Yara M. Rose, Trevor S. Penix, Janice B. Fung, Alexis L. Vanitski, Christian J. Goossen, Spencer G. Bradshaw, Spencer M. Lopp, Aaron D. Pennington, Vincent M. Darmohray, KayLee K. Steiner, Gina E. Kersey, Karl B. Brylow, Mariel V. Pridmore, Joseph S. Hedges, Patrick Rynkiewicz, André O. Hudson

**Affiliations:** a Thomas H. Gosnell School of Life Sciences, Rochester Institute of Technology, Rochester, New York, USA; University of California, Riverside

## Abstract

Here, we report the isolation, identification, and whole-genome sequences of 12 bacterial strains associated with four mushroom species. The study was done as an inquiry-based exercise in an undergraduate genomics course (BIOL 340) in the Thomas H. Gosnell School of Life Sciences at the Rochester Institute of Technology.

## ANNOUNCEMENT

The microbiomes of fungi are understudied due to the fact that the majority of data collected thus far are related to agriculturally relevant species and pathogens (1–3). As genomic techniques are further refined, the ability to study more complex microbial communities can offer vital insights into fungal interactions with the native microflora (4, 5). The overarching goal of this study was to isolate and identify, through whole-genome sequencing, bacteria that associate with mushrooms. This was done as an experiential learning exercise in a genomics course at the Rochester Institute of Technology.

Wild mushrooms were collected from wood surfaces from Black Creek Park (Monroe County, NY; 43.0721°N, 77.8076°W) on 1 September 2019 and identified using the *Mushrooms of Northeast North America* field guide by George Barron (6). The mushroom samples were cut using a sterile scalpel and were used to inoculate Luria broth (LB), Reasoner’s 2A (R2A) broth, potato dextrose (PD) broth, and tryptic soy broth (TSB). Cultures were grown for 24 to 72 h at 25°C with continuous shaking at 150 rpm. Ten-fold serial dilutions were performed, and dilutions in the range of 10^−6^ to 10^−9^ were plated onto the corresponding agar media of LB, R2A, potato dextrose agar (PDA), and tryptic soy agar (TSA) and were incubated for 24 h at room temperature. Colonies were subsequently streaked on the respective media on which they were first grown for purity and were initially chosen for further analyses based on color, size, shape, morphology, and texture.

Using the Qiagen DNeasy UltraClean microbial kit, genomic DNA was isolated from single colonies grown overnight in 5 ml of broth (LB for strains RIT691, RIT692, RIT702, RIT710, RIT711, and RIT714; TSB for RIT693, RIT694, and RIT713; PD for RIT697; and R2A for RIT712). A PCR was performed using the forward primer 341F (5′-CCTACGGGNGGCWGCAG-3′), the reverse primer 805R (5′-GACTACHVGGGTATCTAATCC-3′), and GoTaq green to amplify the variable 3 (V3) and V4 regions of the 16S rRNA gene. The PCR conditions used were as follows: 1 cycle at 95°C for 5 min, followed by 30 cycles at 95°C for 1 min, 55°C for 1 min, and 72°C for 1 min, and ending with one cycle at 72°C for 10 min. The samples were then held at 4°C. Amplicons were sequenced via the Sanger method using the 341F primer. The 16S V3/V4 nucleotide sequences were used to identify the genera using BLAST searches prior to whole-genome sequencing.

For whole-genome sequencing, 0.2 ng/μl of genomic DNA was fragmented and indexed using a Nextera XT library prep kit per the manufacturer’s instructions (Illumina, San Diego, CA) in the genomics core facility in the School of Life Sciences at the Rochester Institute of Technology. The fragment size range was assessed using a DNA 1000 kit on an Agilent 2100 bioanalyzer. Average fragment size (800 bp) was combined with Qubit DNA concentration to determine the molarity of sequencing-ready libraries. Samples were normalized to a final loading concentration of 10 pM and sequenced using the Illumina MiSeq v3 600-cycle run cartridge with 2 × 250-bp paired-end reads. A total yield of 2.34 × 10^7^ reads was obtained, with an average read length of 231.9 bp after trimming with MiSeq default parameters. The sequences were assembled using Unicycler (v0.4.8.0) within Galaxy (v19.09.rc1), filtering out contigs shorter than 200 bp (7, 8). QUAST (v5.0.2) was used to generate statistics on the final assemblies (9). Assembled genomes were taxonomically classified using the Type Strain Genome Server (TYGS) tool (10). Assemblies were submitted as whole-genome shotgun (WGS) sequencing projects to GenBank for annotation using the NCBI Prokaryotic Genome Assembly Pipeline to look for open reading frames (ORFs), rRNAs, and tRNAs (11). Please note that default parameters were used for all software unless otherwise noted.

### Data availability.

Annotation details for each isolate, including accession numbers, are reported in Table 1.

**TABLE 1:** Sequencing and annotation information for each bacterial isolate

Strain	Source	Organism^*a*^	Genome size (bp)	No. of contigs	Genome coverage (×)	*N*_50_ (bp)	GC content of assembly (%)	WGS accession no.	SRA accession no.	No. of ORFs	No. of tRNAs	No. of rRNAs
RIT691	Trichaptum sp.	Unc. Enterobacteriaceae sp.	4,706,300	32	105.65	365,280	54.25	WJYM00000000	SRR10513281	4,291	76	3
RIT692	*Trichaptum* sp.	Unc. *Enterobacteriaceae* sp.	5,194,327	40	81.09	643,341	54.7	WJYL00000000	SRR10513282	4,671	67	3
RIT693	*Trichaptum* sp.	Unc. *Enterobacteriaceae* sp.	5,043,199	51	94.78	260,932	54.25	WJYK00000000	SRR10513273	4,611	75	4
RIT694	*Trichaptum* sp.	Bacillus sp.	5,770,030	162	75.91	72,920	35.15	WJYJ00000000	SRR10513274	5,758	63	6
RIT697	*Trichaptum* sp.	Unc. *Enterobacteriaceae* sp.	5,448,265	48	52.64	294,827	54.59	WJYI00000000	SRR10513275	4,886	70	2
RIT698	Tubaria sp.	Acinetobacter guillouiae	4,576,832	132	111.04	71,764	38.16	WJYH00000000	SRR10513276	4,200	66	2
RIT702	*Tubaria* sp.	Unc. *Enterobacteriaceae* sp.	5,100,156	47	111.53	498,055	54.71	WJYG00000000	SRR10513277	4,579	69	3
RIT710	Tyromyces sp.	Pantoea agglomerans	4,740,543	34	79.32	413,689	55.29	WJYF00000000	SRR10513278	4,329	69	3
RIT711	*Tyromyces* sp.	Unc. *Enterobacteriaceae* sp.	5,113,332	116	118.47	205,802	49.5	WJYE00000000	SRR10513279	4,771	81	4
RIT712	Craterellus sp.	Raoultella sp.	5,315,152	39	101.14	501,597	56.03	WJYD00000000	SRR10513280	4,907	76	4
RIT713	*Craterellus* sp.	Ewingella americana	5,046,485	44	55.64	225,444	53.81	WJYC00000000	SRR10513283	4,628	73	3
RIT714	*Craterellus* sp.	Unc. *Enterobacteriaceae* sp.	4,491,469	40	92.98	299,161	53.57	WJYB00000000	SRR10513284	4,119	74	5

a Unc., uncultured.
